# The development of a comparison approach for Illumina bead chips unravels unexpected challenges applying newest generation microarrays

**DOI:** 10.1186/1471-2105-10-186

**Published:** 2009-06-18

**Authors:** Daniela Eggle, Svenja Debey-Pascher, Marc Beyer, Joachim L Schultze

**Affiliations:** 1 Molecular Tumor Biology and Tumor Immunology, Department of Internal Medicine I, University of Cologne, Kerpener Str. 62, 50924 Cologne, Germany; 2 Genomics and Immunoregulation, Institute for Life and Medical Sciences, University of Bonn, 53155 Bonn, Germany

## Abstract

**Background:**

The MAQC project demonstrated that microarrays with comparable content show inter- and intra-platform reproducibility. However, since the content of gene databases still increases, the development of new generations of microarrays covering new content is mandatory. To better understand the potential challenges updated microarray content might pose on clinical and biological projects we developed a methodology consisting of *in silico *analyses combined with performance analysis using real biological samples.

**Results:**

Here we clearly demonstrate that not only oligonucleotide design but also database content and annotation strongly influence comparability and performance of subsequent generations of microarrays. Additionally, using human blood samples and purified T lymphocyte subsets as two independent examples, we show that a performance analysis using biological samples is crucial for the assessment of consistency and differences.

**Conclusion:**

This study provides an important resource assisting investigators in comparing microarrays of updated content especially when working in a clinical or regulatory setting.

## Background

The ability to assess genome-wide transcriptional profiles of cells, tissues or even whole organs is a cornerstone of the advances genomics has brought to the life and medical sciences [1,2]. DNA microarrays are the major technology used for this purpose [3]. Both in biology and medicine, important new findings have been revealed by this technology [4-6]. More recently, the MicroArray Quality Control (MAQC) project, a community-wide effort initiated and led by FDA (US Food and Drug Administration) scientists, has made a significant contribution assuring reliability and consistency of DNA microarray technology [7-12] at a time when concerns about repeatability, reproducibility and comparability of microarray results were raised [13-15]. The major message from the MAQC project is that microarrays with comparable content show inter- and intra-platform reproducibility of gene expression measurements. Major regulatory agencies such as the FDA or the European Medicine Agencies (E MEA) have recognized genomic technologies, particularly gene expression profiling by DNA microarrays, as opportunities in advancing personalized medicine [16,17]. Therefore, the results established by MAQC are very promising for the use of DNA microarrays in drug development, medical diagnostics and risk assessment, and the use of these technologies has been encouraged by the regulatory agencies.

However, as already outlined by the MAQC project, an important aspect of DNA microarray technology needs further attention [10]. Advances in array technology as well as improvements of genomic database content will lead to the development of new generations of microarrays in upcoming years [18,19]. The currently available annotation of transcripts represented on DNA microarrays (microarray content) is still incomplete. In fact, our knowledge about gene expression is far from being complete, which is reflected by a continuous increase of content of gene databases such as RefSeq [20]. Therefore there have been advances in updating the annotation of microarray probes to the most up-to-date annotation available by providing either new annotation files or software tools for re-annotating existing microarray formats [21-25]. So far, using the most recent DNA microarray technology has always been seen as an advantage – especially when searching for novel transcripts [26]. However, this might be different in the context of drug development, medical diagnostics or risk assessment, where signatures rather than single genes are of highest relevance. Here, unaltered gene annotation and probe sequence content are needed for long-term applications. The potential impact of advances in technology and database content on successfully established diagnostic gene signatures (e.g. the 70-gene signature established by van't Veer *et al*. for predicting therapy outcome in breast cancer patients [27,28]) has not been fully appreciated. It is therefore mandatory to develop approaches and methods that allow fast and decisive assessment of the global impact of database improvements, content changes of microarrays and technical advances.

## Results

### Significant dynamics of gene sequence content of current genome databases

**Figure 1:**
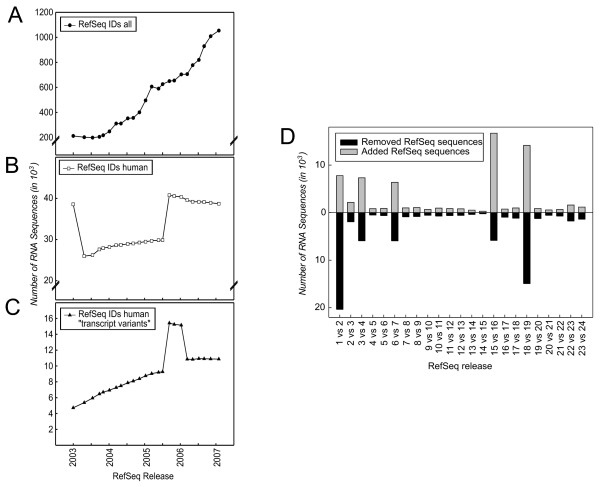
**Dynamics of RefSeq database**. Release statistics retrieved from  shows the development of the RefSeq database, including (A) all RefSeq IDs, (B) human RefSeq IDs, and (C) human RefSeq IDs termed "transcript variant". (D) For human RefSeq IDs, consecutive releases were compared to each other to determine changes in the database over time.

One of the major resources for genomic research are databases such as RefSeq [20], Unigene [29], Ensembl [30], or GenBank [31]. To investigate the underlying dynamics of these databases we performed analyses on both the Refseq and the Ensembl databases. Plotting the official release statistics of the RefSeq database shows a continuing growth of RefSeq sequences (Figure 1A) mainly explained by constant addition of new species. To determine the development of the content of human gene sequences, human database entries (huDE) from the RefSeq release catalog were extracted. Starting with almost 40,000 huDE in release one (R1) the content dropped to less than 28,000 huDE, steadily increased to 30,000 huDE (R16) after which almost 11,000 huDE were added in R17. Since then the overall number of huDE remained stable (Figure 1B). The increase of huDE observed from R2 to R17 can be explained by new knowledge concerning transcript variants (mainly splice variants), which have been added continuously to the database and have more than doubled since 2003 (Figure 1C). Assessing the RefSeq content of subsequent releases (Figure 1D) revealed a surprisingly high number of changes. When performing this analysis on the Ensembl database a similar picture occurred. Since 2004 the number of human entries in the Ensembl database has continuously grown (see Additional file 1A) with a high number of additions and removals of sequences in between subsequent releases (see Additional file 1B). Based on these unexpected and still high dynamics of database content, we hypothesized that the broadly applied microarray technologies, for which RefSeq and Ensembl are two of the main repositories, would be strongly influenced by such changes.

### Content and annotation of microarrays depends on the repository database

**Figure 2:**
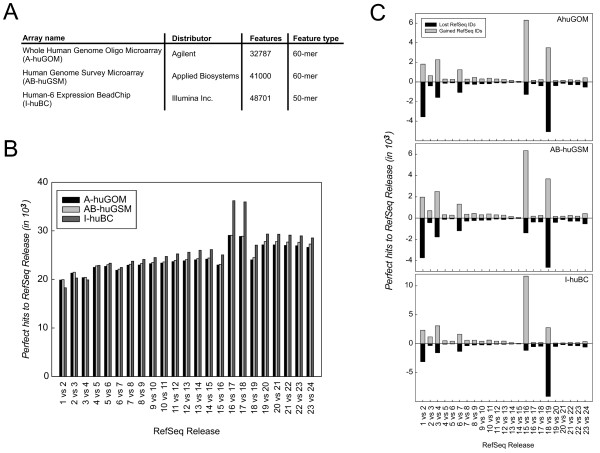
**Influence of Refseq database content on annotation of microarray probes**. (A) Array type, feature type and number of features interrogated by three commercially available oligonucleotide-based microarray platforms. (B) Influence of RefSeq version on annotation of probes used by the three microarray platforms. (C) Differences in the annotation status based on differences of consecutive Refse versions q for the A-huGOM, the AB-huGSM and the I-huBC.

To address the influence of database content on array design and probe content, we used the RefSeq database as a model and first assessed the impact of different RefSeq releases on array annotation. Here, we define array annotation as the number of RefSeq hits obtained by all probes on a microarray. As examples for microarray annotation we used three commercially available oligonucleotide-based microarray platforms, the Whole Human Genome Oligo Microarray distributed by Agilent (A-huGOM), the Human Genome Survey Microarray distributed by Applied Biosystems (AB-huGSM) and the Human BeadChip distributed by Illumina (I-huBC) (Figure 2A) [32-34]. For this analysis the most recent versions of the respective microarrays were used. All oligonucleotide probes on each microarray were blasted against RefSeq releases R1 to R24 to determine the number of RefSeq hits for the respective releases. As shown in Figure 2B, the number of common RefSeq hits between two subsequent releases remained constant for all three platforms except for the increase between R16 and R18 (also seen in Figure 1B). Similarly, when investigating gains and losses of RefSeq hits (Figure 2C) the observed pattern reflected the underlying database changes (Figure 1D).

### Consistency of consecutive array versions strictly depends on database content and annotation

**Figure 3:**
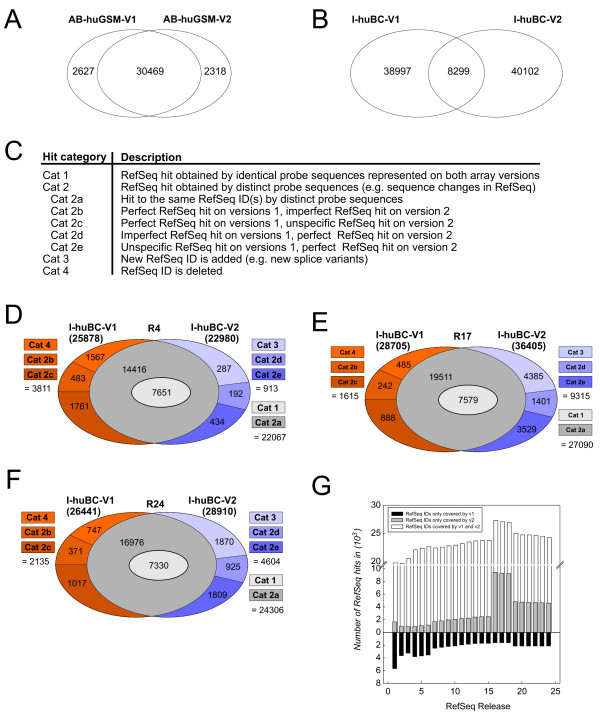
**Comparison of probe level content on subsequent array versions**. For (A) the AB-huGSM and (B) the I-huBC two subsequent array versions were compared regarding their probe level content. (C) Generally, probe sequence changes on consecutive array versions can lead to different numbers and types of RefSeq hits in both array versions. We categorized RefSeq hits resulting from probe sequence changes into 4 hit categories. I-huBC-V1 and I-huBC-V2 were investigated regarding these hit categories based on the following RefSeq releases: (D) R4, (E R17, and (F) R24. (G) Concordances and differences in probe level content between I-huBC-V1 and I-huBC-V2 over all RefSeq releases.

Due to the high dynamics in database content and subsequent annotation changes we were particularly interested in characterizing the impact of database content on subsequent array versions. We therefore further investigated the AB-huGSM and the I-huBC arrays. Both distributing companies recently launched a second version of their original product: AB-huGSM-V2 (January 2005) and I-huBC-V2 (December 2006). The AB-huGSM arrays are comprised of 33,096 (AB-huGSM-V1) and 32,787 (AB-huGSM-V2) oligonucleotides. 30,469 oligonucleotides remained identical between AB-huGSM-V1 and AB-huGSM-V2, whereas 2,627 were removed and 2,318 were added (Figure 3A). The I-huBC arrays included 47,296 (I-huBC-V1) and 48,701 (I-huBC-V2) probes, respectively [34], but to our surprise, only 8,299 oligonucleotides remained identical between I-huBC-V1 and I-huBC-V2 (Figure 3B). We postulated that the dramatic differences concerning probe content would greatly challenge comparability of results. To address this issue in detail, we assessed the overall magnitude of changes using I-huBC-V1 (version 1) and I-huBC-V2 (version 2) as a model. Refseq was used as the annotation database, since both I-huBC-V1 and I-huBC-V2 were designed based on Refseq. We performed a BLAST analysis on all oligonucleotide sequences from both arrays using three Refseq releases (R4, R17 and R24) and categorized hits into one of the 4 categories presented in Figure 3C (see also Additional file 2). R4 represents the release at the time of I-huBC-V1 design (Figure 3D), R17 the release at the time of I-huBC-V2 array design (Figure 3E), and R24 the most current release (Figure 3F). For R17 (Figure 3E) we obtained the highest number of perfect hits for I-huBC-V2 (36,405) as well as the highest number of common RefSeq hits between I-huBC-V1 and I-huBC-V2 (27,090). Also the lowest number of removals (categories 4, 2b and 2c) as well as the highest number of additions (categories 3, 2d and 2e) was obtained. Surprisingly, these numbers changed dramatically when performing the BLAST analysis on the most recent release R24 (Figure 3F), reflecting the strong dependence of array content on database content. The analysis based on R4 (Figure 3D) showed the least agreement in probe level content, as well as the lowest gain of content and the highest number of removals. When running the BLAST analysis on all official RefSeq releases (R1 to R24) we detected the optimum of concordance at R16 and R17 (Figure 3G and Additional file 3), the existent releases at the time of array design of I-huBC-V2. To ensure the reliability of our results we performed the identical analysis on the Ensembl database. Here we also saw differing concordances between I-huBC-V1 and I-huBC-V2 depending on the release. However, we did not observe the drastic difference between the release at the time of array design and the most current release, which might indicate a more stable annotation within Ensembl (see Additional file 1C).

Altogether, comparability of consecutive array versions even on a single platform is a function of oligonucleotide design, database content and annotation available at the time of array design. Unexpectedly, optimal comparability is not achieved with the newest annotation of the RefSeq database but rather with the annotation available at the time of design of the newest array version. As long as the database content is not yet finalized, updates in array design are mandatory to correctly reflect genomic content.

### Selection of representative data sets for best investigation of performance issues

**Figure 4:**
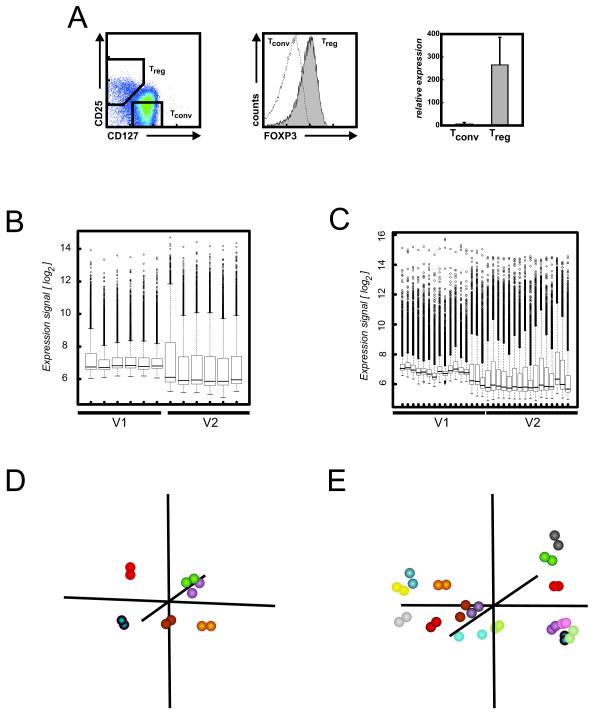
**Technical replication on subsequent array versions**. (A) Experimental analysis for the T_reg _data set: FACS analysis and sorting windows of CD4^+ ^CD127^low ^CD25^+ ^T_reg _cells and CD4^+ ^CD127^+ ^CD25^- ^T_conv _cells (*left*). Expression of FOXP3 in the respective T cell subsets was assessed by flow cytometry (*middle*) and quantitative RT-PCR (*right*). Boxplots were used to compare the dynamic range of signal intensities on the arrays for (B) the T_reg _data set and (C) the whole blood data set. Only signals for the 8299 identical oligonucleotides were used. Technical replicates were checked both by principle component analysis based on the 100 most variable genes for (D) the T_reg _data set and (E) the whole blood data set as well as hierarchical cluster analysis (see Additional file 11).

The above described *in silico *analysis of consecutive array designs is an important first step to estimate the overall impact on array performance. However, we postulate that site-by-site comparison of performance of consecutive array versions by applying biological experiments is the most critical part of future array development as well as compatibility analysis for long-term projects spanning the life time availability of different array versions. We propose that these experiments fulfill specified criteria (see Additional file 4). We performed two different sets of experiments. As an example for a biological screening experiment we compared CD25^+ ^CD127^- ^regulatory T cells (T_reg_, n = 3) as a specialized T cell subpopulation to so-called CD25^- ^CD127^+ ^conventional T cells (T_conv_, n = 3) (Figure 4A) [35,36]. Intracellular staining with FOXP3 mAbs confirmed that CD25^+ ^CD127^- ^cells were indeed T_reg _cells. Moreover, quantitative RT-PCR for FOXP3 mRNA revealed high level expression of FOXP3 in CD25^+ ^CD127^- ^T_reg _cells but not in CD25^- ^CD127^+ ^T_conv _cells (Figure 4A). As an experiment within a diagnostic setting we chose the analysis of peripheral blood samples derived from patients with either scleroderma (n = 11) or bacteremia (n = 7). These samples are part of a larger study addressing diagnostic signatures of systemic diseases in peripheral blood (S. Debey-Pascher, unpublished results). For these samples, we performed microarray analysis on both array types.

### I-huBC-V2 outperforms I-huBC-V1 concerning sensitivity, signal-to-noise-ratio and dynamic range

For further analyses concerning performance issues of two different array versions we cross-annotated the re-blasted probes from the I-huBC-V1 and the I-hu-V2 arrays BC (see Additional file 2). To quickly assess improvement of performance by newer generation technology, we assessed 4 parameters describing important quality aspects, (1) the percentage of detected transcripts reflecting sensitivity, (2) the dynamic range of signal intensities, (3) the values of background/noise signals reflecting signal-to-noise ratio and (4) technical replication reflecting reproducibility. In the T_reg _data set, on average 23.9% of all probes were called present on I-huBC-V1 and 31.0% on I-huBC-V2. Similarly, in the whole blood data set, we obtained mean percentages of present calls of 23.2% for I-huBC-V1 and 30.7% for I-huBC-V2 samples (see Additional file 5). Additionally, probes with low signal intensities on both arrays were generally more often called present on I-huBC-V2 in comparison to I-huBC-V1 suggesting that I-huBC-V2 has a higher detection sensitivity (see Additional file 2, see Additional file 6). Boxplots were used to compare the dynamic range of signals between I-huBC-V1 and I-huBC-V2. When plotting the signals of the 8,299 probes that were identical on both versions, we observed an enlargement of the dynamic range as well as a decrease in median signal intensities on I-huBC-V2 for both data sets (Figure 4B, C) which was due to reduced overall background values on I-huBC-V2 (for cross-annotated probes see Additional file 7A for the T_reg _dataset, see Additional File 7B for the whole blood dataset). Analysis of identical oligonucleotides represented on both versions in conjunction with the use of the same cRNA samples, can be used to assess the performance of both arrays concerning technical replication. When comparing raw signal intensities of such technical replicates we observed increased signal intensities for moderate to highly expressed transcripts on I-huBC-V2 (see Additional file 7C). For visualization we used pairwise scatterplots, principal components analysis (PCA) and hierarchical clustering on normalized data. Samples of the T_reg _data set showed a mean correlation of 0.97 ± 0.005 (see Additional file 8 for a table of all correlations and Additional file 9 for scatterplots) and samples of the whole blood data set a mean correlation of 0.91 ± 0.17 (see Additional file 10 for a table of all correlations and Additional file 11 for scatterplots). These results were confirmed when performing PCA using the 100 most variable probes out of the 8,299 identical oligonucleotides (Figure 4D, E). Additionally, when performing hierarchical clustering on these samples, almost all technical replicates clearly clustered next to each other (see Additional file 12).

### Rank correlation metric reveals significant differences between subsequent microarray versions

**Figure 5:**
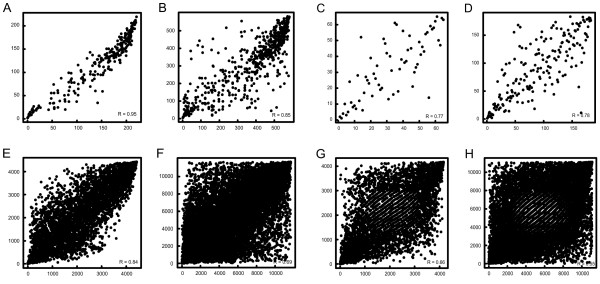
**Comparison of rank correlation of probes between subsequent array versions**. Rank correlation was used as a metric to investigate comparability of hybridization results between the two array versions. In a first step only moderately to highly expressed probes (signal intensity > 500) were used for comparison. This analysis was performed for (A) identical oligonucleotides in the T_reg _data set, (B) cross-annotated probes in the T_reg _data set, (C) identical oligonucleotides in the whole blood data set, and (D) cross-annotated probes in the whole blood data set. In the second step all probes which were present in either one of the sub-groups were used. Again, this analysis was performed for (E) identical oligonucleotides in the T_reg _data set, (F) cross-annotated probes in the T_reg _data set, (G) identical oligonucleotides in the whole blood data set, and (H) cross-annotated probes in the whole blood data set.

To examine the comparability of results across platforms we performed a rank correlation metric [10] and used the ratio of differential expression (between defined groups, here T_reg _versus T_conv _resp. systemic sclerosis versus bacteremia samples) as a basis for ranking. In a first step we used transcripts, which were moderately to highly expressed (signal intensity > 500) in either one of the sub-groups of the data sets to eliminate possible impairment due to absent or low expressed transcripts. Figure 5A shows the result of the analysis based on the 8,299 identical oligonucleotides in the T_reg _data set. Here, 252 transcripts were highly expressed throughout the data set and obtained a rank correlation of 0.95. When using the cross-annotated probes (628) the rank correlation dropped slightly to 0.85 (Figure 5B), which can most probably be ascribed to the differences in oligonucleotide placement within a gene (e.g. closer to 5'end). To our surprise, this high comparability could not be achieved for the whole blood data set. Here, we obtained a rank correlation of 0.77 for identical oligonucleotides (99, Figure 5C) and 0.78 for cross-annotated probes (269, Figure 5D). In a second step we used probes called present in either one of the sub-groups. Within the T_reg _data set, we observed a rank correlation of 0.84 for the identical oligonucleotides and a rank correlation of only 0.69 for the cross-annotated probes (Figure 5E, F). Using the whole blood data set, the rank correlations dropped to 0.66 for the identical oligonucleotides and to only 0.55 for the cross-annotated probes (Figure 5G, H). To examine the strong decrease in rank correlation in more detail, we calculated differentially expressed probes between scleroderma and bacteremia samples for I-huBC-V1 and determined the corresponding signal values on I-huBC-V2 (see Additional file 13). Here, we detected several probes, which were called differentially expressed on I-huBC-V1, but not on I- huBC-V2 due to very low signal values in both sub-groups. Due to the higher detection sensitivity of I-huBC-V2, these probes were not called absent. To rule out that this difference was intrinsic to the whole blood samples we performed the same analysis for the T_reg _data set. Similar to the whole blood data set, several probes showing differential expression on I-huBC-V1 were not called differentially expressed on I-huBC-V2 and also had low signal values for both T cell sub-groups (see Additional file 14). Among these probes was also FOXP3, which is the most important marker of T_reg _cells. As shown in Figure 4A, differential expression of FOXP3 between T_reg _and T_conv _cells was already confirmed by quantitative RT-PCR as well as intracellular FACS analysis to assess protein expression. Therefore, at least for FOXP3, the data generated with I-huBC-V1 reflected real differences between the tested sub-groups while the I-huBC-V2 did not. Furthermore, BLAST analysis of the FOXP3 probes revealed distinct, yet perfect hits (100% identity), for both I-huBC-V1 and I-huBC-V2 (data not shown), suggesting that a functional probe was exchanged by a non-functional.

**Figure 6:**
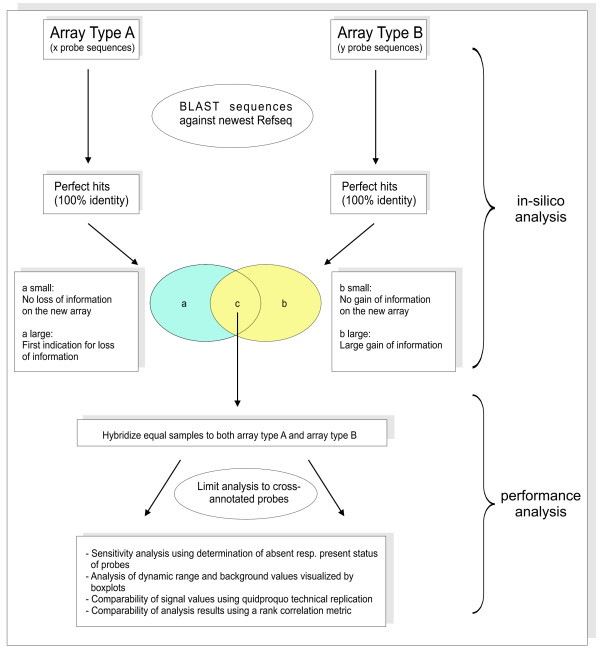
**Workflow diagram**. Proposed method to quickly determine the impact of changes between subsequent microarray versions. This generalized impact analysis consists of an *in silico *analysis combined with an experimental performance analysis.

We therefore propose a comparison approach combining an extended *in silico *analysis with the experimental analysis (Figure 6). The *in silico *analysis consists of re-blasting all probe sequences, collecting perfect hits, and categorization of hits. The experimental analysis should include at least cross-annotation, analysis of sensitivity, dynamic range, technical replication and a rank correlation metric. The global impact of upgrading microarray technology and content on any given project can be quickly estimated by this standardized approach.

## Discussion

Most recently, validity and comparability of transcriptional profiling using different microarray platforms has been very elegantly demonstrated by the MAQC consortium [10]. Proving consistency of these technologies when introducing technological advances was suggested by MAQC as a major issue for future development. Here we have addressed the overall impact of improvements of genomic database content and annotation over time and the impact of technology optimization on major performance issues of a typical microarray analysis. Unexpectedly, database content and annotation as exemplified for the Refseq database still remains highly dynamic, which by itself has a significant impact on microarray probe annotation. Using an *in silico *approach based on BLAST analysis combined with categorization of probes and respective cross-annotation approaches, we demonstrate that content changes on a given microarray platform are also influenced by database dynamics. Moreover, we conducted a performance analysis combining common quality control measures with a rank correlation metric and show that the inclusion of real biological experiments is mandatory to estimate the overall impact of technology improvements on data consistency. Using the Illumina BeadChip platform as an example, we demonstrate that a large change of probe content between subsequent array versions results in incompatible data in addition to unexpected challenges, such as significant introduction of non-functional probes. This has high impact on biological screening experiments, when signals for known marker genes are lost (as exemplified for FOXP3). Even higher impact can be expected for experiments within a diagnostic setting, where content and technology changes will lead to incompatible diagnostic signatures. Up to now, using the most recent DNA microarray format has always been seen as an advantage, since the most recent version is usually an improvement of the old version. However, this might only be true for the technical performance of an array.

It should be noted that we chose the Illumina BeadChip over the Agilent arrays as an example, since the number of changes between subsequent array generations was significantly higher for this platform. Also, we have only used ~20,000 cross-annotated probes for performance analysis, which is less than 50% of the content. The reason for this strictness was, in part, based on a recent publication by Lee et al. demonstrating high signal disagreement for probes targeting genes susceptible to alternative splicing [37]. We therefore limited our analysis to probes with identical targets.

As already outlined by the MAQC project, high throughput technologies including microarrays for transcriptional profiling require significantly more attention to quality control and comparability than any test measuring only a single data point [10]. The MAQC project clearly demonstrated that comparability of microarray technology is already high 1) when restricting the analysis to a comparable set of data points (genes) and 2) when comparing high throughput technologies developed approximately at the same time. Here we clearly show that a next important step in genomic sciences will be to quickly introduce standardized general impact analyses to assess newer generation technologies. It would be desirable to introduce the presented approach as a starting point for further projects within the MAQC consortium. Next steps could be to test the overall impact of the presented approach in the larger consortium and perform such impact analyses on a grand scale respectively when new technologies become available again.

## Conclusion

In summary, standardized methods and approaches are critically needed to quickly address the impact of introducing upgrades of high throughput technologies on project content.

## Methods

### Retrieving database releases and statistics

Human sequences for RefSeq releases 1 through 24 (September 2007) were obtained in two steps. First, the human RefSeq entries for each release were extracted from the release catalog which can be obtained from . Second, by using GI numbers and the E-utilities provided by NCBI, fasta sequences for each entry were downloaded. All fasta sequences for a Release were stored in a separate file. Human sequences for Ensembl releases 21–52 (April 2004 – December 2008) were obtained as fasta sequences from .

### BLAST analysis of probes

For performing the BLAST analyses we used the Standalone BLAST tool (v2.2.16) distributed by NCBI . Probe sequences for the different array versions were extracted from the annotation files provided by the manufacturers and fasta files were generated from them. For blasting probe sequences we used the blastn program. The output file (tab-delimited) was imported into R for further analysis. Three different classes of hits to the databases can a be retrieved for each probe: (1) a hit was called 'perfect' if the alignment length was equal to the probe length and returned a 100% identity, (2) a hit was called 'imperfect' if the alignment length was equal to the probe length and returned an identity which was 90% < identity < 100% and (3) a hit was called 'unspecific' if the alignment length was shorter than the probe length.

### Cross-annotation of probes

By BLAST analysis a set of probes was identified with perfect hits to Refseq. For cross-annotation purposes three types of probes with perfect hits have **to **be considered: (1) probes showing a single perfect hit to one Refseq ID, (2) probes with hits to more than one Refseq ID, all of which are splice variants of the same gene and (3) probes showing hits to more than one Refseq comprising different genes. To ensure cross-annotation of probes only within one probe type we chose the following cross-annotation approach:

Let *list*(*X*_*A*_) (*list*(*Y*_*B*_)) be the list of Refseq IDs with a perfect hit of probe *X *(*Y*) on arrays *A *(*B*). Then *X *and *Y *will be cross-annotated if *list *(*X*_*A*_) = *list *(*Y*_*B*_). This approach ensures cross-annotation of probes within one probe type.

### Determination of absent or present status of individual genes

For comparing the absent or present status of transcripts on the I-huBC-V1 and the I-huBC-V2 array, respectively, the following criteria were used: A probe was called present on a single array, if the detection p-value < 0.05. A probe was called present within a sub-group, if it was called present in at least 2/3 of the samples within this sub-group. Otherwise it was called absent.

### Data analysis

Raw data collection for Illumina BeadChip arrays was performed using Illumina BeadStudio software. All data analysis was performed using the R Statistical language [38] and packages from the Bioconductor [39] project. Data sets were normalized using the quantile normalization method implemented in the 'affy' package. Hierarchical clustering was performed using the 'hcluster' package with average linkage and Pearson's correlation as the linkage resp. distance methods. Principal components analysis was performed using the pcurve package. Pairwise scatterplots for investigating technical replication were performed on normalized data. When performing an analysis based on the 8,299 identical probes data from I-huBC-V1 and I-huBC-V2 was limited to these 8,299 proes and then normalized together using quantile normalization. For all other analyses based on cross-annotated probes, data was normalized individually within each array version, since a combined normalization across cross-annotated probes (in contrast to identical probes) could potentially alter the results.

Differentially expressed genes were calculated using Student's t-test using the following criteria: fold change > 1.75, p-value < 0.05 and difference of mean group-signal > 100. Variation of probes across a data set was determined using the variation coefficient for each probe (mean/stdev) across all samples. The 100 most variable probes were then used for further analysis.

### Rank correlation metric

To examine the comparability of results from two different array versions we performed a rank correlation comparison. Cross-annotated probes that were moderately to highly expressed (signal intensity > 500) or present in one of the sub-groups on either one array type were used for analysis. Probes were ranked according to the following criteria: (1) log fold change, (2) p-value and (3) difference of means. Rank correlations were calculated using Pearson's correlation coefficient implemented in R.

### Sample collection and preparation

Blood samples from patients with systemic sclerosis or bacteremia, respectively, were collected in PAXgene blood RNA tubes (BD Biosciences, Heidelberg, Germany) after written informed consent had been obtained and following approval by the institutional review board. CD4^+ ^CD127^low ^CD25^+ ^(T_reg_) and CD4^+ ^CD127^+ ^CD25^-^(T_conv_) T cells were stained with CD4, CD25 and CD127 mAb (all from BD Pharmingen) and sorted on a FACSDiva cell sorter. Cell purity after isolation was assessed by intracellular staining for FOXP3 (e-bioscience) and routinely showed purities >95%.

### RNA preparation and microarray hybridization

RNA from T_reg _and T_conv _cells lysed in TRIzol (Invitrogen, Karlsruhe, Germany) was isolated according to the manufacturer's protocol with subsequent column purification using the RNeasy MinElute Cleanup Kit (Qiagen, Hilden, Germany). Total RNA from PAXgene samples was prepared according to the manufacturer's recommendations including an optional DNAse digestion step. cDNA and biotin-labeled cRNA synthesis was generated from 100 ng total RNA using the Illumina^® ^TotalPrep™ RNA Amplification Kit (Applied Biosystems, Darmstadt, Germany). cRNA (1.5 μg) was hybridized to Human-6 Expression BeadChips V1 and V2 (Illumina, San Diego, CA) and scanned on Illumina BeadStation 500×. All microarray data has been submitted to Gene Expression Omnibus (GSE16031).

## Authors' contributions

DE carried out the analyses, drafted and wrote the manuscript. SD contributed to the analyses and performed the microarray experiments. MB contributed the biological experiments for the Treg data set. JLS conceived of the study, participated in its design and coordination and drafted the manuscript. All authors read and approved the final manuscript.

## Supplementary Material

Additional file 1 **Dynamics of the Ensembl database**. (A) Release statistics retrieved from  shows the development of the Ensembl database for all human entries. (B) Consecutive releases were compared to each other to determine changes in the database over time. (C) Concordances and differences in probe level content between I-huBC-V1 and I-huBC-V2 over all Ensembl releases. Click here for file

Additional file 2 **Supplemental methods**. A summary of all supplemental methods Click here for file

Additional file 3 **Optimum analysis**. Optimum analysis for array concordance Click here for file

Additional file 4 **Criteria for data sets**. 4 different criteria for representative data sets. Click here for file

Additional file 5 **Present calls**. Number of present probes for each sample in both the Treg and the whole blood dataset Click here for file

Additional file 6 **Status of probes**. Absent resp. present status of probes Click here for file

Additional file 7 **Dynamic range of signal intensities for cross-annotated probes**. Boxplots can not only be used to determine the distribution of intensity signals across a single array but to compare the dynamic range of signals in between two arrays. Here, we used this quality measurement to compare the subset of cross-annotated probes. Depicted are boxplots showing the dynamic range of cross-annotated probe signals for (A) the T_reg _data set and (B) the whole blood data set. (C) Example of a comparison of raw signal intensities for a technical replicate. Click here for file

Additional file 8 **Correlations T_reg_**. Correlations of technical replicates in T_reg _data set Click here for file

Additional file 9 **Correlation of technical replicates in the T_reg _data set**. To investigate the outcome of technical replication we used pairwise scatterplots. For perfect technical replicates one would expect a straight diagonal line in a pairwise scatterplot. Data for both array versions was limited to 8,299 identical oligonucleotides. Pairwise scatterplots of signal intensities were performed on the normalized T_reg _set. Shown are scatterplots for samples 1–6 (A-F). Click here for file

Additional file 10 **Correlations whole blood**. Correlations of technical replicates in whole blood data set Click here for file

Additional file 11 **Correlation of technical replicates in the whole blood data set**. To investigate the outcome of technical replication we used pairwise scatterplots. For perfect technical replicates one would expect a straight diagonal line in a pairwise scatterplot. Data for both array versions was limited to 8,299 identical oligonucleotides. Pairwise scatterplots of signal intensities were performed on the normalized whole blood data set. Shown are scatterplots for samples 1–16. Click here for file

Additional file 12 **Hierarchical cluster analysis of technical replicates**. To investigate the outcome of technical replication we used pairwise scatterplots, principal components analysis (PCA) and hierarchical clustering on normalized data. For perfect technical replication one would expect a side-by-side clustering of replicated samples when using PCA (see Figure 4D, E) or a clustering approach. Hierarchical cluster analysis was performed on normalized data using the 100 most variable genes in both data sets. (A) In the T_reg _data set T_reg _samples are denoted in orange, T_conv _samples are denoted in black. (B) In the whole blood data set scleroderma samples are denoted in orange, bacteremia samples are denoted in black. The naming convention in both data sets is as follows: sample type_sample id_array version. Click here for file

Additional file 13 **Differentially expressed genes whole blood**. Differentially expressed genes (FC > 1.75, p-value < 0.05, diff > 100) between Scleroderma and Bacteremia samples on I-huBC-V1 and corresponding values for these genes on I-huBC-V2 Click here for file

Additional file 14 **Differentially expressed genes T_reg_**. Differentially expressed genes (FC > 1.75, p-value < 0.05, diff > 100) between T_reg _and non-T_reg _samples on I-huBC-V1 and corresponding values for these genes on I-huBC-V2 Click here for file

## References

[B1] Pennacchio LA, Rubin EM (2001). Genomic strategies to identify mammalian regulatory sequences. Nat Rev Genet.

[B2] Reinke V, White KP (2002). Developmental genomic approaches in model organisms. Annu Rev Genomics Hum Genet.

[B3] Schena M, Shalon D, Davis RW, Brown PO (1995). Quantitative monitoring of gene expression patterns with a complementary DNA microarray. Science.

[B4] Bild AH, Yao G, Chang JT, Wang Q, Potti A, Chasse D, Joshi MB, Harpole D, Lancaster JM, Berchuck A, Olson JA, Marks JR, Dressman HK, West M, Nevins JR (2006). Oncogenic pathway signatures in human cancers as a guide to targeted therapies. Nature.

[B5] Golub TR, Slonim DK, Tamayo P, Huard C, Gaasenbeek M, Mesirov JP, Coller H, Loh ML, Downing JR, Caligiuri MA, Bloomfield CD, Lander ES (1999). Molecular classification of cancer: class discovery and class prediction by gene expression monitoring. Science.

[B6] Lim LP, Lau NC, Garrett-Engele P, Grimson A, Schelter JM, Castle J, Bartel DP, Linsley PS, Johnson JM (2005). Microarray analysis shows that some microRNAs downregulate large numbers of target mRNAs. Nature.

[B7] Canales RD, Luo Y, Willey JC, Austermiller B, Barbacioru CC, Boysen C, Hunkapiller K, Jensen RV, Knight CR, Lee KY, Ma Y, Maqsodi B, Papallo A, Peters EH, Poulter K, Ruppel PL, Samaha RR, Shi L, Yang W, Zhang L, Goodsaid FM (2006). Evaluation of DNA microarray results with quantitative gene expression platforms. Nat Biotechnol.

[B8] Guo L, Lobenhofer EK, Wang C, Shippy R, Harris SC, Zhang L, Mei N, Chen T, Herman D, Goodsaid FM, Hurban P, Phillips KL, Xu J, Deng X, Sun YA, Tong W, Dragan YP, Shi L (2006). Rat toxicogenomic study reveals analytical consistency across microarray platforms. Nat Biotechnol.

[B9] Patterson TA, Lobenhofer EK, Fulmer-Smentek SB, Collins PJ, Chu TM, Bao W, Fang H, Kawasaki ES, Hager J, Tikhonova IR, Walker SJ, Zhang L, Hurban P, de Longueville F, Fuscoe JC, Tong W, Shi L, Wolfinger RD (2006). Performance comparison of one-color and two-color platforms within the MicroArray Quality Control (MAQC) project. Nat Biotechnol.

[B10] Shi L, Reid LH, Jones WD, Shippy R, Warrington JA, Baker SC, Collins PJ, de Longueville F, Kawasaki ES, Lee KY, Luo Y, Sun YA, Willey JM, Setterquist RA, Fischer GM, Tong W, Dragan YP, Dix DJ, Frueh FW, Goodsaid FM, Herman D, Jensen RV, Johnson CD, Lobenhofer EK, Puri RK, Schrf U, Thierry-Mieg J, Wang C, Wilson M, Wolber PK, Zhang L, Slikker W, Shi L, Reid LH (2006). The MicroArray Quality Control (MAQC) project shows inter- and intraplatform reproducibility of gene expression measurements. Nat Biotechnol.

[B11] Shippy R, Fulmer-Smentek S, Jensen RV, Jones WD, Wolber PK, Johnson CD, Pine PS, Boysen C, Guo X, Chudin E, Sun YA, Willey JC, Thierry-Mieg J, Thierry-Mieg D, Setterquist RA, Wilson M, Lucas AB, Novoradovskaya N, Papallo A, Turpaz Y, Baker SC, Warrington JA, Shi L, Herman D (2006). Using RNA sample titrations to assess microarray platform performance and normalization techniques. Nat Biotechnol.

[B12] Tong W, Lucas AB, Shippy R, Fan X, Fang H, Hong H, Orr MS, Chu TM, Guo X, Collins PJ, Sun YA, Wang SJ, Bao W, Wolfinger RD, Shchegrova S, Guo L, Warrington JA, Shi L (2006). Evaluation of external RNA controls for the assessment of microarray performance. Nat Biotechnol.

[B13] Irizarry RA, Warren D, Spencer F, Kim IF, Biswal S, Frank BC, Gabrielson E, Garcia JG, Geoghegan J, Germino G, Griffin C, Hilmer SC, Hoffman E, Jedlicka AE, Kawasaki E, Martinez-Murillo F, Morsberger L, Lee H, Petersen D, Quackenbush J, Scott A, Wilson M, Yang Y, Ye SQ, Yu W (2005). Multiple-laboratory comparison of microarray platforms. Nat Methods.

[B14] Kuo WP, Liu F, Trimarchi J, Punzo C, Lombardi M, Sarang J, Whipple ME, Maysuria M, Serikawa K, Lee SY, McCrann D, Kang J, Shearstone JR, Burke J, Park DJ, Wang X, Rector TL, Ricciardi-Castagnoli P, Perrin S, Choi S, Bumgarner R, Kim JH, Short GF, Freeman MW, Seed B, Jensen R, Church GM, Hovig E, Cepko CL, Park P, Ohno-Machado L, Jenssen TK (2006). A sequence-oriented comparison of gene expression measurements across different hybridization-based technologies. Nat Biotechnol.

[B15] Larkin JE, Frank BC, Gavras H, Sultana R, Quackenbush J (2005). Independence and reproducibility across microarray platforms. Nat Methods.

[B16] Frueh FW (2006). Impact of microarray data quality on genomic data submissions to the FDA. Nat Biotechnol.

[B17] Lesko LJ, Woodcock J (2004). Translation of pharmacogenomics and pharmacogenetics: a regulatory perspective. Nat Rev Drug Discov.

[B18] Hardiman G (2006). Microarrays Technologies 2006: an overview. Pharmacogenomics.

[B19] Hoheisel JD (2006). Microarray technology: beyond transcript profiling and genotype analysis. Nat Rev Genet.

[B20] Pruitt KD, Tatusova T, Maglott DR (2007). NCBI reference sequences (RefSeq): a curated non-redundant sequence database of genomes, transcripts and proteins. Nucleic Acids Res.

[B21] Dai M, Wang P, Boyd AD, Kostov G, Athey B, Jones EG, Bunney WE, Myers RM, Speed TP, Akil H, Watson SJ, Meng F (2005). Evolving gene/transcript definitions significantly alter the interpretation of GeneChip data. Nucleic Acids Res.

[B22] de Leeuw WC, Rauwerda H, Jonker MJ, Breit TM (2008). Salvaging Affymetrix probes after probe-level re-annotation. BMC Res Notes.

[B23] Ferrari F, Bortoluzzi S, Coppe A, Sirota A, Safran M, Shmoish M, Ferrari S, Lancet D, Danieli GA, Bicciato S (2007). Novel definition files for human GeneChips based on GeneAnnot. BMC Bioinformatics.

[B24] Harbig J, Sprinkle R, Enkemann SA (2005). A sequence-based identification of the genes detected by probesets on the Affymetrix U133 plus 2.0 array. Nucleic Acids Res.

[B25] Berg BH van den, Konieczka JH, McCarthy FM, Burgess SC (2009). ArrayIDer: automated structural re-annotation pipeline for DNA microarrays. BMC Bioinformatics.

[B26] Classen S, Zander T, Eggle D, Chemnitz JM, Brors B, Buchmann I, Popov A, Beyer M, Eils R, Debey S, S chultzeJL (2007). Human resting CD4+ T cells are constitutively inhibited by TGF beta under steady-state conditions. J Immunol.

[B27] Vijver MJ van de, He YD, van't Veer LJ, Dai H, Hart AA, Voskuil DW, Schreiber GJ, Peterse JL, Roberts C, Marton MJ, Parrish M, Atsma D, Witteve A, Glas en A, Delahaye L, Velde T van der, Bartelink H, Rodenhuis S, Rutgers ET, Friend SH, Bernards R (2002). A gene-expression signature as a predictor of survival in breast cancer. The New England journal of medicine.

[B28] van't Veer LJ, Dai H, Vijver MJ van de, He YD, Hart AA, Mao M, Peterse HL, Kooy K van der, Marton MJ, Witteveen AT, Schreiber GJ, Kerkhoven RM, Roberts C, Linsley PS, Bernards R, Friend SH (2002). Gene expression profiling predicts clinical outcome of breast cancer. Nature.

[B29] Pontius J, Wagner L, Schuler G (2003). UniGene: a unified view of the transcriptome. The NCBI Handbook.

[B30] Flicek P, Aken BL, Beal K, Ballester B, Caccamo M, Chen Y, Clarke L, Coates G, Cunningham F, Cutts T, Down T, Dyer SC, Eyre T, Fitzgerald S, Fernandez-Banet J, Graf S, Haider S, Hammond M, Holland R, Howe KL, Howe K, Johnson N, Jenkinson A, Kahari A, Keefe D, Kokocinski F, Kulesha E, Lawson D, Longden I, Megy K, Meidl P, Overduin B, Parker A, Pritchard B, Prlic A, Rice S, Rios D, Schuster M, Sealy I, Slater G, Smedley D, Spudich G, Trevanion S, Vilella AJ, Vogel J, White S, Wood M, Birney E, Cox T, Curwen V, Durbin R, Fernandez-Suarez XM, Herrero J, Hubbard TJ, Kasprzyk A, Proctor G, Smith J, Ureta-Vidal A, Searle S (2008). Ensembl 2008. Nucleic acids research.

[B31] Benson DA, Karsch-Mizrachi I, Lipman DJ, Ostell J, Wheeler DL (2006). GenBank. Nucleic acids research.

[B32] Avery OT, MacLeod CM, McCarty M (1979). Studies on the chemical nature of the substance inducing transformation of pneumococcal types. Inductions of transformation by a desoxyribonucleic acid fraction isolated from pneumococcus type III. J Exp Med.

[B33] Kronick MN (2004). Creation of the whole human genome microarray. Expert review of proteomics.

[B34] Kuhn K, Baker SC, Chudin E, Lieu MH, Oeser S, Bennett H, Rigault P, Barker D, McDaniel TK, Chee MS (2004). A novel, high-performance random array platform for quantitative gene expression profiling. Genome Res.

[B35] Liu W, Putnam AL, Xu-Yu Z, Szot GL, Lee MR, Zhu S, Gottlieb PA, Kapranov P, Gingeras TR, Fazekas de St Groth B, Clayberger C, Soper DM, Ziegler SF, Bluestone JA (2006). CD127 expression inversely correlates with FoxP3 and suppressive function of human CD4+ T reg cells. J Exp Med.

[B36] Seddiki N, Santner-Nanan B, Martinson J, Zaunders J, Sasson S, Landay A, Solomon M, Selby W, Alexander SI, Nanan R, Kelleher A, Fazekas de St Groth B (2006). Expression of interleukin (IL)-2 and IL-7 receptors discriminates between human regulatory and activated T cells. J Exp Med.

[B37] Lee JC, Stiles D, Lu J, Cam MC (2007). A detailed transcript-level probe annotation reveals alternative splicing based microarray platform differences. BMC Genomics.

[B38] R Development Core Team (2007). R: A Language and Environment for Statistical Computing.

[B39] Gentleman RC, Carey VJ, Bates DM, Bolstad B, Dettling M, Dudoit S, Ellis B, Gautier L, Ge Y, Gentry J, Hornik K, Hothorn T, Huber W, Iacus S, Irizarry R, Leisch F, Li C, Maechler M, Rossini AJ, Sawitzki G, Smith C, Smyth G, Tierney L, Yang JY, Zhang J (2004). Bioconductor: open software development for computational biology and bioinformatics. Genome Biol.

