# Supplementary figures and images for: The development of a comparison approach for Illumina bead chips unravels unexpected challenges applying newest generation microarrays
Source: BMC Bioinformatics. 2009 Jun 18;10:186. doi: 10.1186/1471-2105-10-186 (PMC2711080; doi:10.1186/1471-2105-10-186)

A

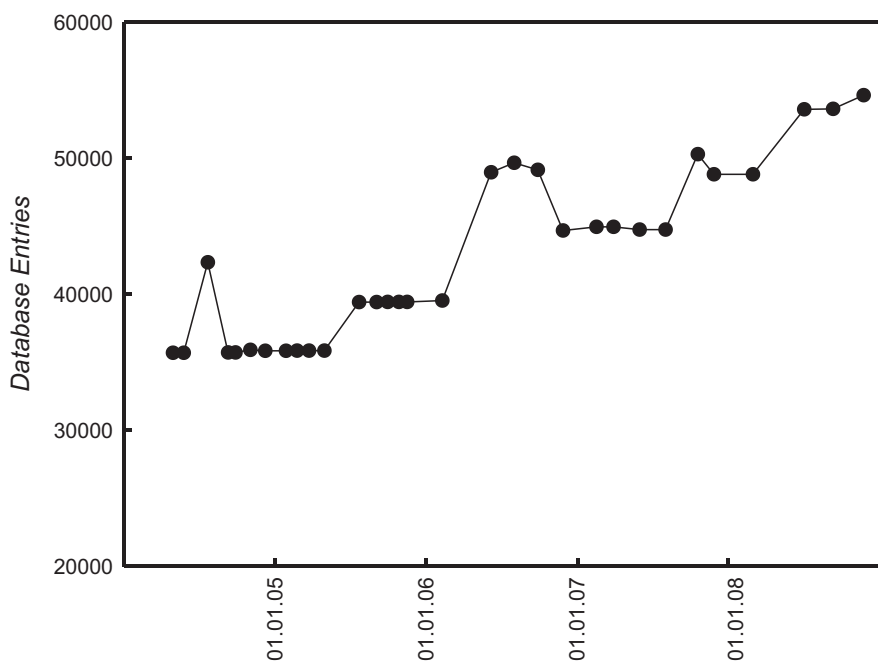

B

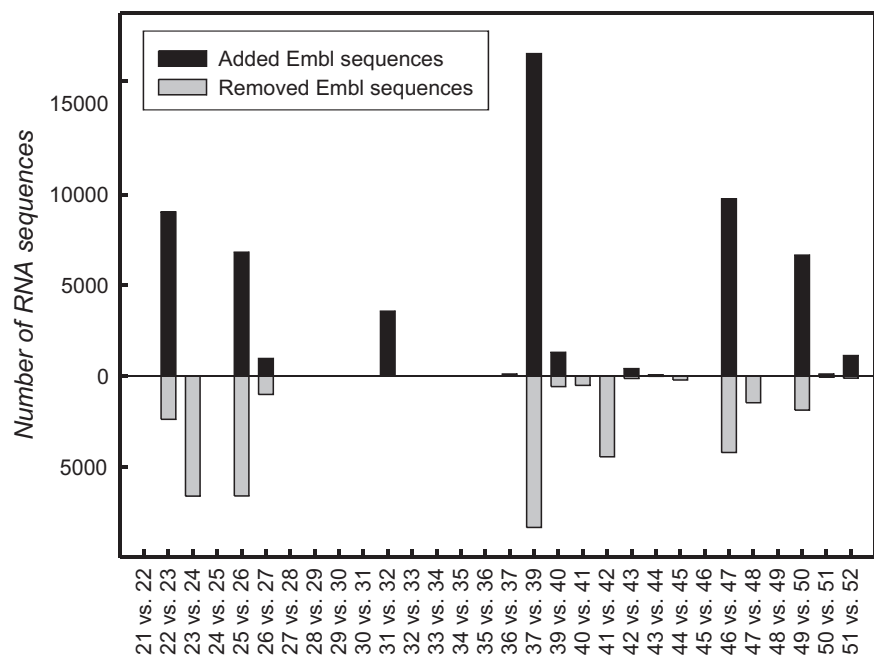

C

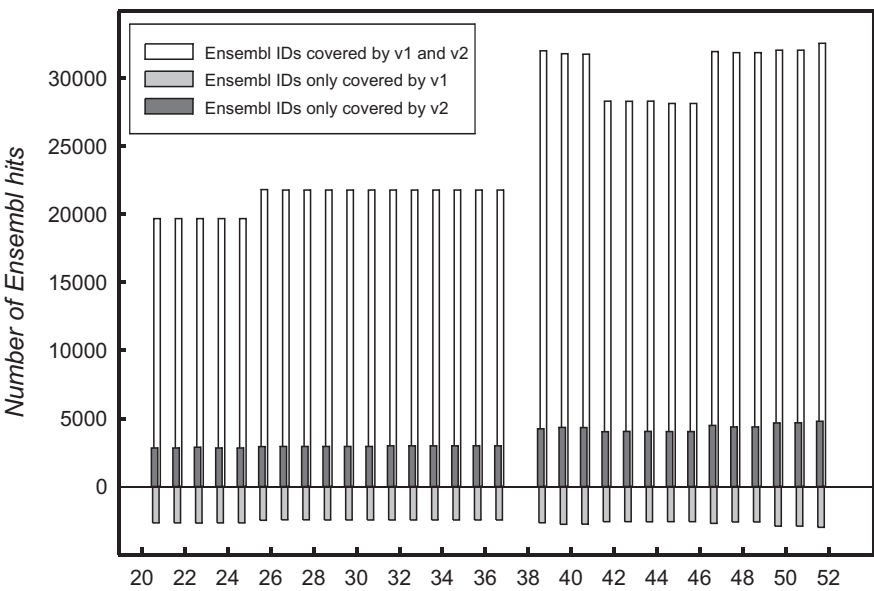

Supplement: Additional file 1 — Dynamics of the Ensembl database. (A) Release statistics retrieved from shows the development of the Ensembl database for all human entries. (B) Consecutive releases were compared to each other to determine changes in the database over time. (C) Concordances and differences in probe level content between I-huBC-V1 and I-huBC-V2 over all Ensembl releases. [file 1471-2105-10-186-S1.pdf]

**A**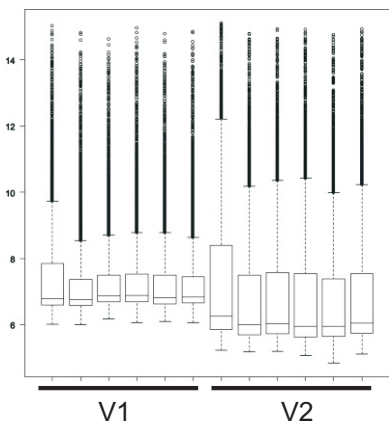**B**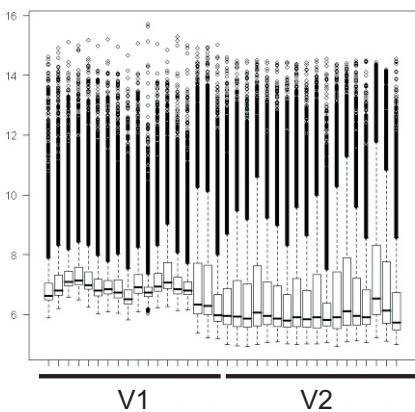**C**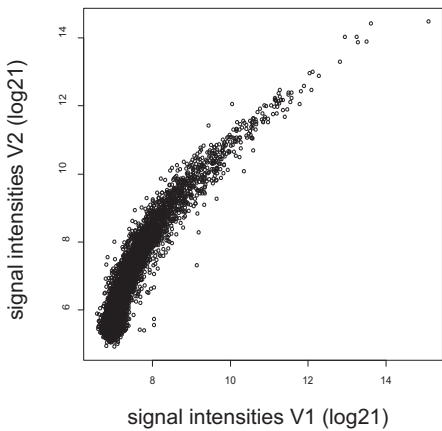

Supplement: Additional file 7 — Dynamic range of signal intensities for cross-annotated probes. Boxplots can not only be used to determine the distribution of intensity signals across a single array but to compare the dynamic range of signals in between two arrays. Here, we used this quality measurement to compare the subset of cross-annotated probes. Depicted are boxplots showing the dynamic range of cross-annotated probe signals for (A) the Treg data set and (B) the whole blood data set. (C) Example of a comparison of raw signal intensities for a technical replicate. [file 1471-2105-10-186-S7.pdf]

**A**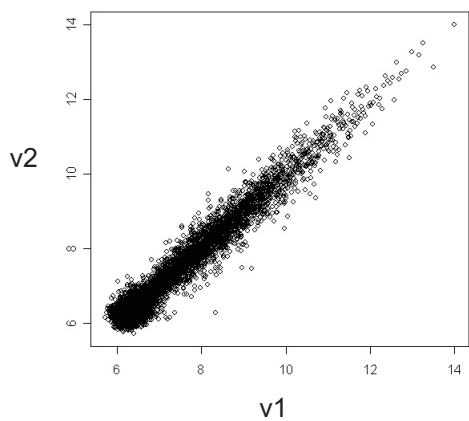**B**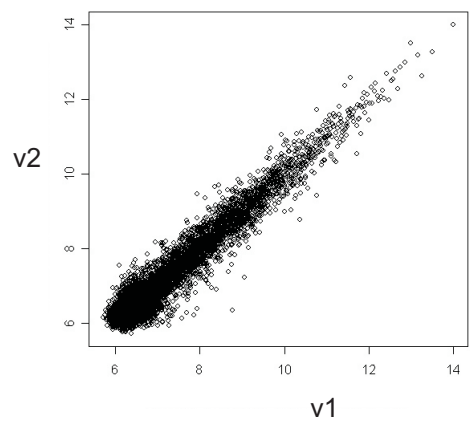**C**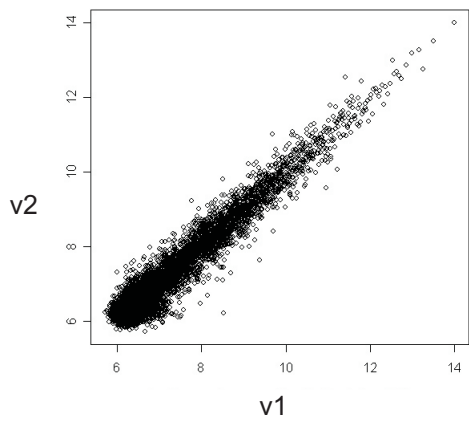**D**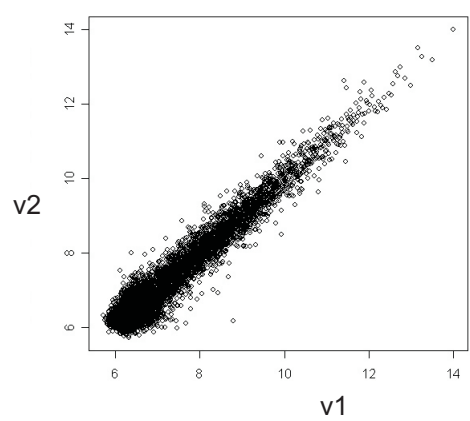**E**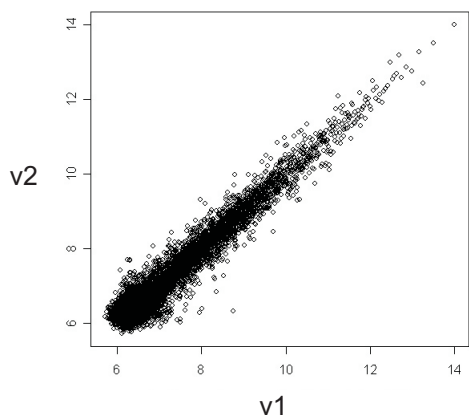**F**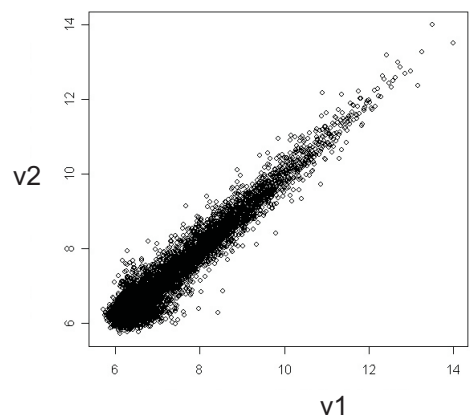

Supplement: Additional file 9 — Correlation of technical replicates in the Treg data set. To investigate the outcome of technical replication we used pairwise scatterplots. For perfect technical replicates one would expect a straight diagonal line in a pairwise scatterplot. Data for both array versions was limited to 8,299 identical oligonucleotides. Pairwise scatterplots of signal intensities were performed on the normalized Treg set. Shown are scatterplots for samples 1–6 (A-F). [file 1471-2105-10-186-S9.pdf]

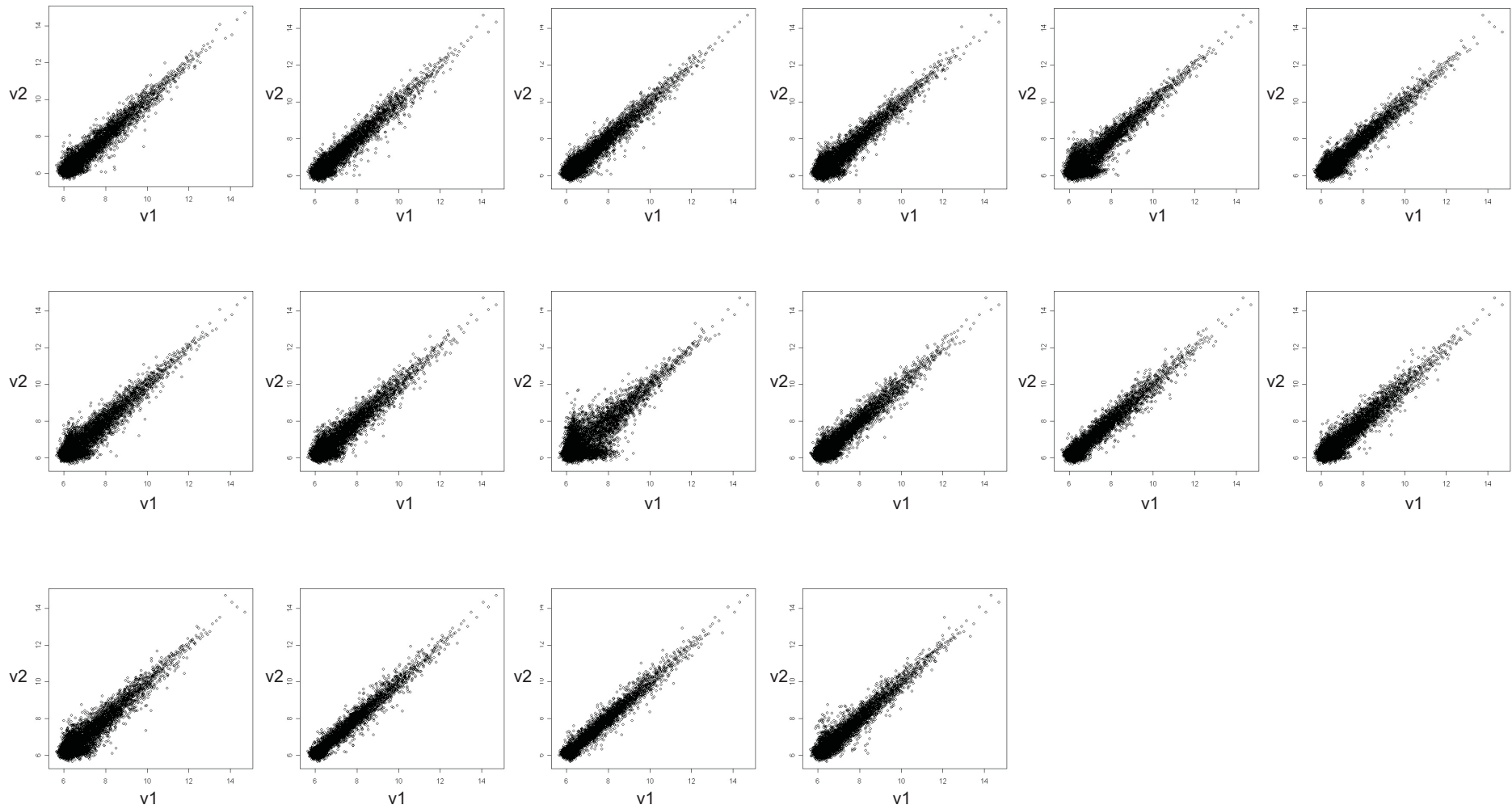

Supplement: Additional file 11 — Correlation of technical replicates in the whole blood data set. To investigate the outcome of technical replication we used pairwise scatterplots. For perfect technical replicates one would expect a straight diagonal line in a pairwise scatterplot. Data for both array versions was limited to 8,299 identical oligonucleotides. Pairwise scatterplots of signal intensities were performed on the normalized whole blood data set. Shown are scatterplots for samples 1–16. [file 1471-2105-10-186-S11.pdf]

A

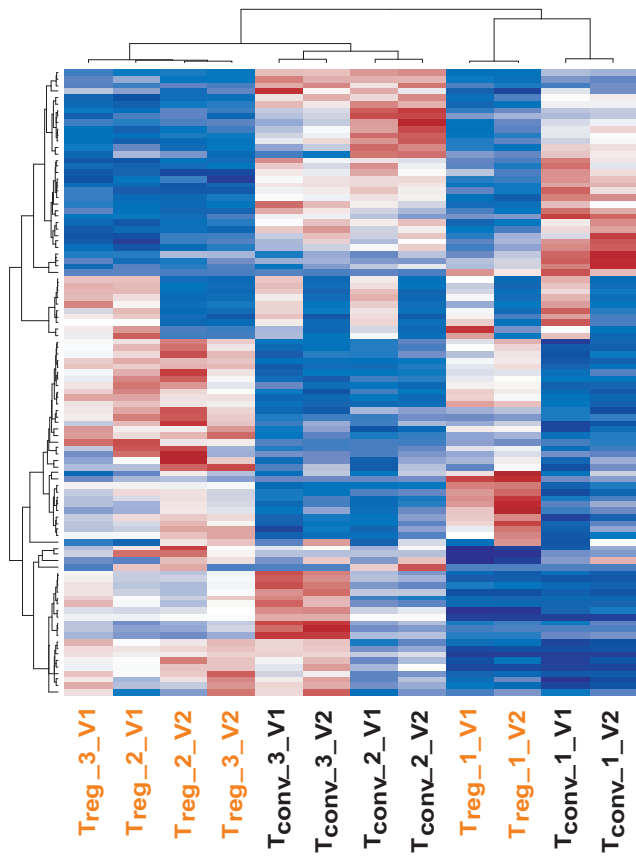

B

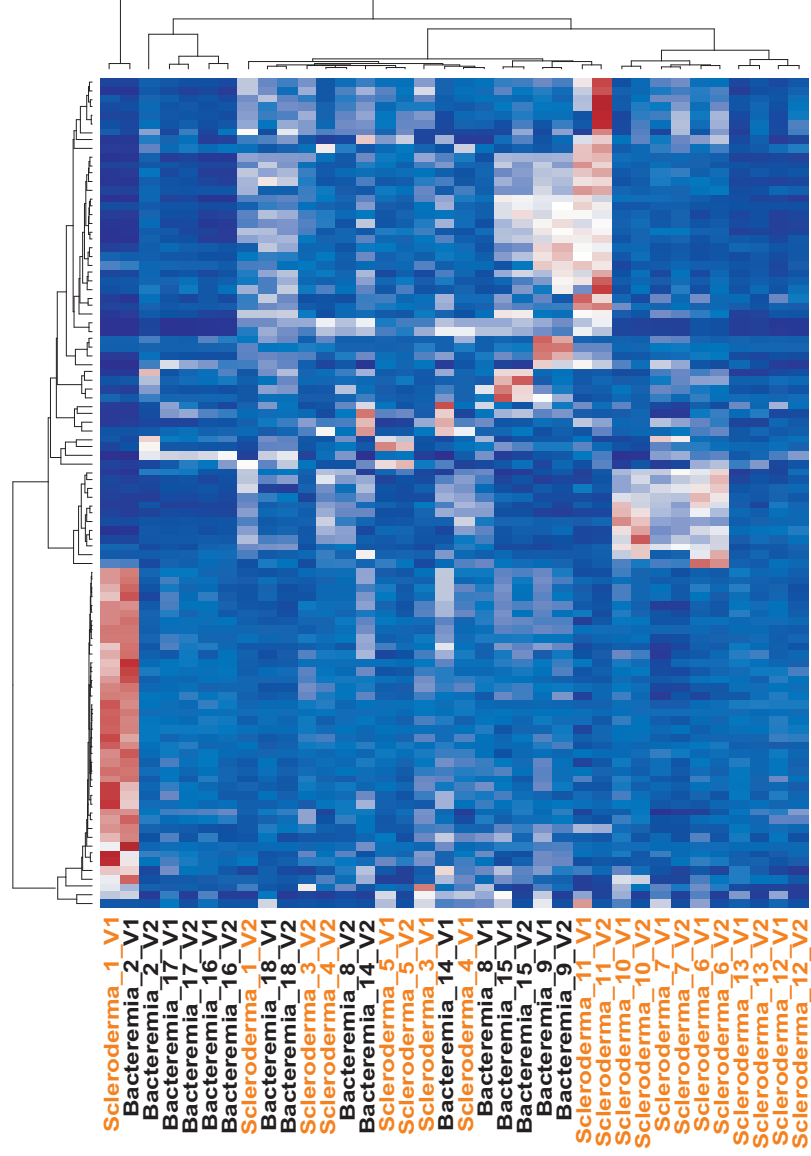

Supplement: Additional file 12 — Hierarchical cluster analysis of technical replicates. To investigate the outcome of technical replication we used pairwise scatterplots, principal components analysis (PCA) and hierarchical clustering on normalized data. For perfect technical replication one would expect a side-by-side clustering of replicated samples when using PCA (see Figure 4D, E) or a clustering approach. Hierarchical cluster analysis was performed on normalized data using the 100 most variable genes in both data sets. (A) In the Treg data set Treg samples are denoted in orange, Tconv samples are denoted in black. (B) In the whole blood data set scleroderma samples are denoted in orange, bacteremia samples are denoted in black. The naming convention in both data sets is as follows: sample type_sample id_array version. [file 1471-2105-10-186-S12.pdf]
