# Supplementary material for: The development of a comparison approach for Illumina bead chips unravels unexpected challenges applying newest generation microarrays
Source: BMC Bioinformatics. 2009 Jun 18;10:186. doi: 10.1186/1471-2105-10-186 (PMC2711080; doi:10.1186/1471-2105-10-186)
Supplement: Additional file 2 — Supplemental methods. A summary of all supplemental methods [file 1471-2105-10-186-S2.pdf]

Supplemental Methods

Categorization of RefSeq hits

Generally, probe sequence changes on consecutive array versions can lead to different numbers and types of RefSeq hits in both array versions. Types of RefSeq hits include “perfect” (100% sequence identity), “imperfect” (>90% sequence identity) or “unspecific” (<90% sequence identity) hits. Furthermore, number and type of RefSeq hits depend on changes within the RefSeq database.

We categorized RefSeq hits resulting from probe sequence changes as follows:

- Hit Category 1: RefSeq hit obtained by identical probe sequences represented on both array versions
- Hit Category 2: RefSeq hit obtained by distinct probe sequences (sequence changes in RefSeq, design improvement, etc.)
    - Category 2a: Hit to the same RefSeq ID(s) by distinct probe sequences
    - Category 2b: Perfect RefSeq hit on version 1, imperfect RefSeq hit on version 2
    - Category 2c: Perfect RefSeq hit on version 1, unspecific RefSeq hit on version 2
    - Category 2d: Imperfect RefSeq hit on version 1, perfect RefSeq hit on version 2
    - Category 2e: Unspecific RefSeq hit on version 1, perfect RefSeq hit on version 2
- Category 3: New RefSeq is added (e.g. splice variants, prediction (XM_ probe) was correct)
- Category 4: RefSeq is deleted (e.g. prediction (XM_ probe) turned out to be wrong, problems in synthesis, etc.)

Number of cross-annotated probes on consecutive microarrays stays stable

For further analyses concerning performance issues of two different array versions we cross-annotated the re-blasted probes from I-huBC-V1 and the I-huBC-V2 arrays principally using the approach used by the MAQC project[10]. In contrast to the MAQC project, which condensed its mapping to a ‘one-probe-to-one-gene’ approach, we took all perfect hits into account. Therefore, our cross-annotation approach had to consider three types of probes: (1) probes which show a single perfect hit to a Refseq, (2) probes with multiple perfect hits to more than one Refseq which are all splice variants of the same gene and (3) probes which show hits to more than one Refseq comprising different genes. For each probe on the I-huBC-V1 array we compared its list of perfect Refseq hits to all probes on the I-huBC-V2 array. When identifying a probe on the I-huBC-V2 showing an exact match in length and content of the hit list, the two probes were cross-annotated. This approach ensured cross-annotation of probes within one probe type (1 to 3) but also excluded probes of type

(2) which showed multiple hits on both I-huBC-V1 and the I-huBC-V2 but had distinct number of hits for both versions (distinct number of splice variants). In the latter case signals may not be comparable due to different expression profiles of single splice variants which would introduce further variation when investigating comparability of performance of two consecutive microarrays. Using this approach we cross-annotated probes based on all 24 RefSeq releases. We postulated that the number of cross-annotated probes would increase over time due to an increase of previously non-annotated probes hitting Refseq IDs in later versions of Refseq. To our great surprise, the number of cross-annotated probes stayed relatively constant over all releases (see **Figure** below) and did not show the expected increase at R16 and R17. This can be explained by the huge increase in the number of splice variants for the two releases (see **Figure 1C**) as well as the cross-annotation approach itself which prohibits cross-annotation of probes targeting a distinct number of splice variants. For all further analyses we used the cross-annotation based on the latest RefSeq release (R24) and therefore worked on 20,456 probes.

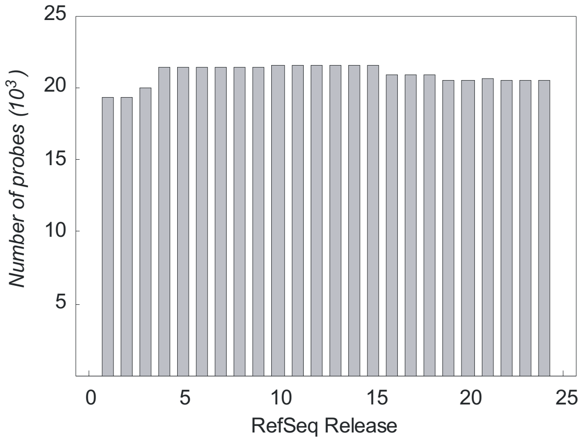

Selection of data sets for best investigation of performance issues

We propose to use representative data sets to assess array performance in (1) a biological screening experiment (e.g. cell type comparison) respectively (2) in a group analysis setting (e.g. clinical sub-classification of diseases), (3) coverage of as many present probes as possible, and (4) availability of validating data for single genes. We performed two different sets of experiments .Overall, the number of probes present in at least one sample (resp. sub-group) on I-huBC-V1 was 28,358 (resp.13,104), representing 72.9% (resp. 46.7%) of cross-annotated. The number of probes present in at least one sample (resp. subgroup) on I-huBC-V2 was 24,986 (resp. 18,096), representing 67.0% (56.0%) of cross-annotated probes. The larger number of probes called present in individual samples on the I-huBC-V1 array is indicative for a higher variability of the earlier array version.

Assessing the percentage of detected transcripts reflecting sensitivity

We further determined the percentage of identical and cross-annotated probes called present on the I-huBC-V1 array, but absent on the I-huBC-V2 array and vice versa for each subgroup in the data sets (**Supplemental Table 4**). As expected from the higher percentage of probes called present on the I-huBC-V2 array, we also saw a significantly higher number of cross-annotated probes present on the I-huBC-V2 array compared to the I-huBC-V1 array (up to 4 fold). Still, there was a small percentage of probes that were present on I-huBC-V1 but absent on I-huBC-V2. Since we observed a rather high variability of probes called present in single samples compared to sub-groups on the I-huBC-V1, we hypothesized that these probes would have very low signal values (just above background value) on the I-huBC-V1 array and would therefore have been false-positively called present on the I-huBC-V1. Indeed, when determining these probes, ~75% showed values close to background level (**Supplemental Table 4**). Altogether, these data further support that the newer generation array technology is of higher sensitivity respectively lower detection limit.

Generalized impact analysis on array performance when upgrading array technologies

The in-silico analysis includes the following steps:

1. Re-blasting of probe sequences from both array types (A and B) using the most up-to-date database annotation.
2. Collecting perfect hits (100% identity) for each probe.
3. Determining the number of hits which are achieved by both array types (“c”) or only by array type A (“a”) or B (“b”), respectively and categorization of hits according to Table 1.

For the subsequent performance analysis individual samples should be hybridized to both array types A and B. The biological samples used for this performance analysis should fulfill the criteria mentioned in Supplemental Table 1.

The experimental analysis includes the following steps:

1. Cross-annotation of data sets generated on arrays to be compared.
2. Sensitivity analysis using determination of absent or present status of probes.
3. Analysis of dynamic range and background values visualized by boxplots.
4. Comparability of signal values using quidproquo technical replication.
5. Comparability of analysis results using a rank correlation metric.
